# The value of Eye Movement Desensitization Reprocessing in the treatment of tinnitus: study protocol for a randomized controlled trial

**DOI:** 10.1186/s13063-018-3121-6

**Published:** 2019-01-09

**Authors:** Tine Luyten, Paul Van de Heyning, Laure Jacquemin, Nancy Van Looveren, Frank Declau, Erik Fransen, Annick Gilles

**Affiliations:** 1 Faculty of Medicine and Health Sciences, Campus Drie Eiken, University of Antwerp, Antwerp, Belgium; 2 University Department of Otorhinolaryngology and Head and Neck Surgery, Antwerp University Hospital, Wilrijkstraat 10, 2650 Edegem, Belgium; 3 Department of Education, Health and Social Work, University College Ghent, Ghent, Belgium; 4 Hoorzorg Van Looveren BVBA, Herentalsebaan 275, 2150 Borsbeek, Belgium

**Keywords:** Tinnitus retraining therapy, Cognitive behavioral therapy, Eye movement desensitization reprocessing, Chronic tinnitus, Event-related potentials

## Abstract

**Background:**

Patients suffering from chronic, subjective tinnitus are on a quest to find a cure or any form of alleviation for their persistent complaint. Current recommended therapy forms provide psychotherapeutic interventions that are intended to train the patient how to deal with the tinnitus sound. Pharmaceutical managements are used to reduce secondary effects of the tinnitus sound such as sleep deprivation, emotional and concentration difficulties, but these treatments do not cure the tinnitus. Recent studies have shown that Tinnitus Retraining Therapy (TRT) significantly improves the quality of life for tinnitus patients. Furthermore, several studies have reported that cognitive behavioral therapy (CBT) relieves a substantial amount of distress by changing dysfunctional cognitions. However, when the tinnitus causes great interference with daily functioning, these treatment methods are not always sufficiently effective. Recent insights show that Eye Movement Desensitization Reprocessing (EMDR) is a highly effective therapy for medically unexplained symptoms such as chronic pain and phantom pain. In scientific research, tinnitus is compared to phantom limb pain. Starting from tinnitus as a phantom percept we therefore aim to demonstrate that the operating mechanisms of EMDR may also be an effective treatment method for patients with subjective tinnitus. The aim of this randomized controlled study with blind evaluator is to examine the effect of EMDR compared to CBT in chronic tinnitus patients.

To our knowledge, there are no other studies that evaluate both methods simultaneously.

**Methods/design:**

A total of 166 patients with subjective, chronic, non-pulsatile tinnitus will be randomized in two treatment groups: TRT + CBT versus TRT + EMDR. The experimental group will receive the bimodal therapy TRT/EMDR and the active control group will receive the bimodal therapy TRT/CBT. Evaluations will take place at baseline before therapy, at the end of the treatment and 3 months after therapy. The score on the Tinnitus Functional Index (TFI) will be used as the primary outcome measurement. Secondary outcome measurements are the Visual Analogue Scale of Loudness (VAS), Tinnitus Questionnaire (TQ), Hospital Anxiety and Depression Scale (HADS), Hyperacusis Questionnaire (HQ), psychoacoustic measurements and event-related potentials (ERP).

**Discussion:**

The objective is to evaluate whether the bimodal therapy TRT and EMDR can provide faster and/or more relief from the annoyance experienced in chronic tinnitus patients’ daily lives compared to the bimodal therapy TRT and CBT.

So far there has been no prospective, randomized controlled, clinical trial with blind evaluator that compares CBT and EMDR as a treatment for tinnitus.

**Trial registration:**

ClinicalTrials.gov, ID: NCT03114878. April 14, 2017.

**Electronic supplementary material:**

The online version of this article (10.1186/s13063-018-3121-6) contains supplementary material, which is available to authorized users.

## Background

Tinnitus is defined as the perception of sound without the presence of an external auditory input and, therefore, it is also referred to as a phantom percept [1]. Usually tinnitus is transient but 8 to 20% of the population suffers from chronic tinnitus. About 1–3% of this population experiences so much distress that they seek medical help [2]. Tinnitus sounds reported by patients are squeaking, buzzing or hissing sounds which can lead to a critical degree of psychological discomfort and can have a serious impact on one’s quality of life. There is a high co-morbidity found in patients suffering from more severe forms of tinnitus, such as depression, insomnia and anxiety [3]. The diagnosis and treatment, therefore, require great expertise to detect the existing etiology and co-morbidities. For the treatment of complex tinnitus cases, a multidisciplinary approach by ENT physicians, specialized audiologists and psychologists is required [4, 5]. Currently there is no pharmaco- or psychotherapeutic treatment that can alleviate chronic, subjective tinnitus. Effective forms of therapy such as Tinnitus Retraining Therapy (TRT) and cognitive behavioral therapy (CBT) are intended to teach patients how to deal with the tinnitus sound either through sound enrichment and psycho-education or through the alteration of cognitions. The clinical management for tinnitus over recent years consists of audiologic interventions and CBT [3, 6]. A stepped-care approach is recommended by guidelines that start with education as first step and end with specialized multidisciplinary CBT as the last step [7]. For a proportion of patients with tinnitus symptoms these treatment forms lead to a significant reduction of distress. When the tinnitus, however, causes a great interference with daily functioning, these treatment methods may not be sufficiently effective [7]. Recent research has shown that EMDR could be an effective therapy for medically unexplained symptoms such as chronic pain and phantom pain [8–12]. Francine Shapiro stated in 2001 that phantom pain can be perceived as a manifestation of the stored somatic memory [13]. The experiencing of pain while the limb is absent is an example of dysfunctional memory storage. Through EMDR treatment these somatic memory and pain sensations can be targeted and restored. Therefore, we hypothesize that EMDR may also be an effective treatment method for patients with subjective tinnitus. Tinnitus is known as phantom sound and previous research has shown overlapping brain networks between tinnitus and phantom pain [1]. Multiple dynamic brain networks such as the perception network, salience network, distress and memory network play a major role in eliciting and maintaining this phantom perception [1, 14, 15]. Previous research has shown that changes (i.e., neural plasticity) in the auditory system elicited by a sound trauma could cause tinnitus. This neural plasticity is a reaction of the nervous system when there is a reorganization and hyperactivity in the auditory cortex causing a tinnitus sensation [16, 17]. Magnetoencephalography (MEG) and electroencephalography (qEEG) earlier showed that different neural networks are at the basis of tinnitus loudness and tinnitus-related distress. Certain types of tinnitus are related to the reorganization and hyperactivity of the auditory central nervous system [18, 19]. Changes in neural activity were also observed in non-auditory brain areas [14]. The stress and emotional reactions associated with tinnitus probably find their neuronal correlate in networks at the level of the amygdala, the hippocampus and anterior cingulate cortex, the parahippocampus and the insula. Research shows that memory mechanisms play an important role in the persistence of the awareness of the phantom percept. Therefore, these mechanisms are inextricably linked to the reinforcement of the associated distress [1]. In these regions neuronal changes occur. We know that tinnitus is a phenomenon in which networks of auditory and non-auditory brain areas influence each other [1, 15, 20–22]. Hence, tinnitus viewed from the perspective of a trauma, more specifically in the auditory and limbic regions, leads to the need for effective information processing. The development of new neural networks could be generated through EMDR: bilateral stimulation promotes the plasticity of the brain causing neural networks to be adjusted. Recent studies demonstrate increase in limbic processing along with decreased frontal activation as a consequence of bilateral stimulation. These patterns of neural activity could facilitate the integration and reintegration of information [23, 24].

The use of bilateral stimuli to treat tinnitus is an innovative treatment method. In the literature, only limited data can be found in a few case studies where EMDR treatment is performed on tinnitus patients [25, 26]. These scarce data, however, provide insufficient insights concerning the EMDR treatment method and the mechanisms. Therefore, a randomized controlled study trial is necessary to examine the effectiveness of EMDR in persons suffering from tinnitus.

### Study objectives

The present study is a prospective, randomized controlled, clinical trial with blind evaluator in order to assess whether EMDR adds value to the treatment in chronic tinnitus patients. The general purpose of the current study is to examine a bimodal therapy for chronic, subjective tinnitus consisting of the combination of the TRT and EMDR. The goal of this treatment is reducing the subjective discomfort of tinnitus and thereby improving the quality of life of the patient.

## Methods/design

**Fig. 1:**
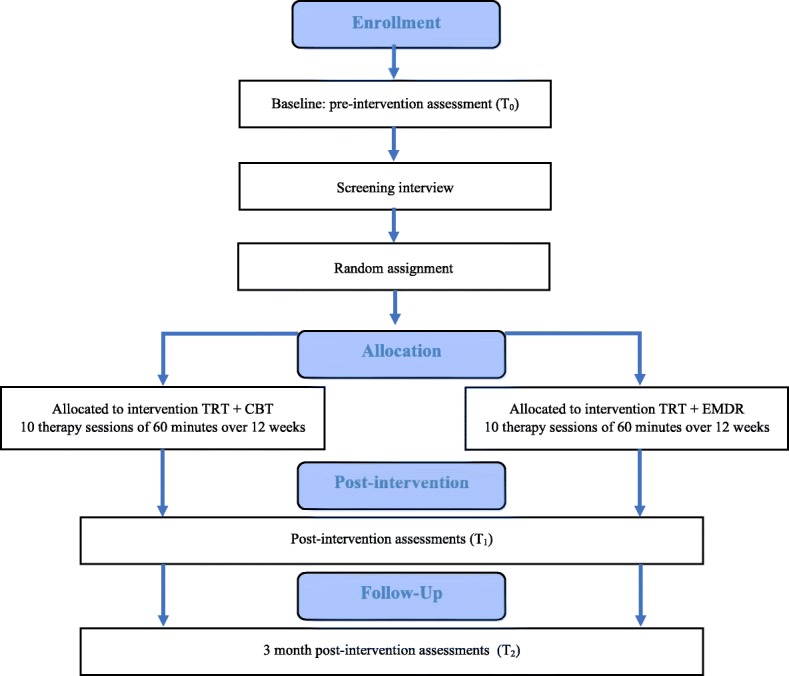
Consolidated Standards of Reporting Trials (CONSORT) study flow diagram. *TRT* Tinnitus Retraining Therapy, *CBT* Cognitive Behavioural Therapy, *EMDR* Eye Movement Desensitization Reprocessing

There will be three test moments during the trial: before the start of therapy (T0), after the treatment sessions (T1) and 3 months after the last therapy session (T2). The primary and secondary outcome measurements will be assessed at every test moment. The difference between bimodal therapy 1 (TRT + CBT) and bimodal therapy 2 (TRT + EMDR) will be assessed. The experimental intervention, namely TRT + EMDR, will be compared to the active control group, namely TRT + CBT, as shown in the Consolidated Standards of Reporting Trials (CONSORT) flow diagram in Fig. 1. The Standard Protocol Items: Recommendations for Interventional Trials (SPIRIT) Checklist was utilized as guideline for this study (Additional file 1).

### Study population

Patients will be referred by the Ear, Nose and Throat (ENT) Department of the Antwerp University Hospital and will be randomized into group 1 (TRT + CBT) or group 2 (TRT + EMDR). The allocation sequence will be determined by the date the patients are referred to the study.

### Inclusion and exclusion criteria

The appropriate ENT and audiological examinations will be performed in order to assess whether a patient meets the inclusion criteria. During the study, the patient has the right to cease the study at any time. The occurrence of severe adverse events can also lead to discontinuation of the study.

The inclusion criteria are as follows:

- Tinnitus type: chronic, subjective, non-pulsatile tinnitus
- Duration of tinnitus = more than 3 months
- Minimum age of the patient is 18 years old – maximum age is 75 years old
- Tinnitus Functional Index (TFI) score ≥ 25 to < 90
- Stable use of medication during therapy

A patient is excluded from the study for the following reasons:

- HADS – score: anxiety and depression subscores > 15
- HQ – score > 40
- Objective, pulsatile tinnitus
- Active middle-ear pathology
- Neurological and psychiatric co-morbidity for which acute psychotherapy is ongoing
- Psychosis, schizophrenia, epilepsy
- Pregnancy

### Study protocol

A number of 166 patients with subjective, chronic, non-pulsatile tinnitus will be randomized in two treatment groups: TRT + CBT versus TRT + EMDR. Licensed therapists will provide all treatments.

#### Tinnitus Retraining Therapy

TRT is a therapy originally developed by Prof. Pawel Jastrebroff and Dr. Jonathan Hazell [27]. During the counseling, the patient is educated about the working mechanism of tinnitus and how to deal with the emotional and physical responses. The main goal is to habituate to the sound of the ringing in the ears. Every patient will receive five 60-min sessions of TRT. The TRT will consist of counseling, sound enrichment and adjustment of a noise masker. A licensed tinnitus therapist will perform the TRT sessions using the patient counseling guideline of Henry et al. [28].

#### Cognitive behavioral therapy

CBT is the combination of behavioral therapy with interventions that have been developed from cognitive psychology. The founders of CBT are Aaron Beck and Albert Ellis [29]. The core idea is the assumption that so-called negative cognitions are responsible for dysfunctional behavior. The techniques used in CBT focus on changing the content of these irrational cognitions. The way tinnitus influences our daily functioning is largely dependent on the meaning we give to this sound and the thoughts we have associated with tinnitus determine which emotional reactions are triggered. CBT helps the patient to change the thoughts associated with tinnitus and as a consequence, alter the emotional response. Mindfulness and a number of relaxation techniques were also developed within CBT. The interventions are based on a 4-year postgraduate training in CBT and a tinnitus master class by Dr.Aazh in 2013. Dr. Hashir Aazh BSc, MSc, PhD focuses on researching and providing specialist therapy for patients experiencing tinnitus [30].

Following treatment outline will be the guideline for the CBT sessions Table 1.

**Table 1:** Intervention based on the cognitive behavioral therapy (CBT) treatment outline

Phase number	Treatment outline
Phase 1	Educating the patient about the auditory system (brief, only if necessary after TRT sessions)
Phase 2	Introduction of CBT and assess the motivation and commitment towards the therapy
Phase 3	Help identify thoughts, emotions and behavior in response to sound
Phase 4	Help identify negative automatic thoughts and core belief
Phase 5	Education about common errors of judgment and distortions in thoughts
Phase 6	Challenging unhelpful thoughts and creating counterstatements
Phase 7	Empirical demonstration: experiencing the consequence of helpful thoughts
Phase 8	Behavioral desensitization and graded exposure

Note: CBT treatment outline by Dr. Aazh based on handouts and personal communication from the Tinnitus and Hyperacusis Master Class in March 2013 at Birkbeck College, London

Every patient who is randomized into the TRT + CBT – group will receive five 60-min sessions of CBT performed by a licensed clinical psychologist and psychotherapist specialized in CBT.

#### Eye Movement Desensitization and Reprocessing

A licensed clinical psychologist will conduct the EMDR sessions according to the original protocol developed by Shapiro in 1987 [31]. This is a scientifically grounded, psychotherapeutic approach that represents a specific method within a wider theoretical model called “Adaptive Information Processing (AIP)” [31]. This term refers to the innate capacity of the brain to process life experiences and incentives to achieve an adaptive solution. The AIP model states that the information processing system of the brain can become obstructed as a result of psychological trauma and that specific methods, such as EMDR, are a catalyst for effective information processing that leads to a rapid and adaptive processing of the trauma [32]. Tinnitus can be interpreted as trauma at the level of the auditory regions of the brain. The development of new neural networks where the tinnitus sound is processed as neutral and non-threatening could be generated through EMDR. Eye movements, tactile stimulation (buzzers) and auditory stimuli (headphone) can be used to facilitate the activation of the left and right side of the brain. Within the treatment this is called bilateral stimulation [10, 31–33].

The trauma-network is stimulated through a combination of standardized procedures that include these repetitive eye movements, auditory signals and tactile vibrations. To induce the stimuli, a validated EMDR tool is used to help the clinician. The EyeScan 4000 will be used in this study to guarantee continuity in the visual, auditory and tactile stimuli that are used to stimulate bilateral activation. The original EMDR protocol developed by Shapiro will be followed in order to guarantee validity and test-retest reliability [34] Table 2.

**Table 2:** Summary of the intervention based on the standard treatment Eye Movement Desensitization Reprocessing (EMDR) protocol by Shapiro (2001) [13]

Phase Number		
Phase 1	Client history	Negative thoughts, feelings, sensations and experiences associated with the complaints will be identified
Phase 2	Preparation phase	State stability of the client Secure a “stop” sign Installation of the calm/safe place
Phase 3	Assessment phase	Target issue, memory, event, or symptom Target image Negative cognition: NC Positive cognition: PC VoC (Validity of Cognition) Emotions SUDs (Subjective Units of Distress) Location of bodily sensation
Phase 4	Desensitization phase	“*Bring the target image and negative cognition to mind, notice where you are feeling it in your body*” Set of bilateral stimuli (BLS) as fast as the client can tolerate After a set: “*What do you get now?*” “*What are you noticing?*” If client reports new material: “*Go with that*”.
Phase 5	Installation phase	“*Do the words PC still fit, or would another positive statement be more suitable?*” Check VoC: “*Think about the original incident and the words PC. How true do they feel now* (1–7)*?*” “*Bring the target image and positive cognition together in your mind.*” Completion of sets of BLS until no change
Phase 6	Body scan	The client will be asked to mentally scan the entire body and report to the therapist what they can feel
Phase 7	Closure	The therapist will end the session by checking if the client feels calm. Gain of the session and reactions that can appear after the session will be discussed
Phase 8	Reevaluation	The client gives an assessment of the results achieved in the follow-up session

Note: Summary based on the standard protocol of Shapiro, F. (2001) [13]. Eye movement desensitization and reprocessing. Basic principles, protocols and procedures (2nd ed.). New York: Guilford Press

The protocol contains eight phases. The first phase is the Client History in which the focus lies on the tinnitus complaints. In this phase the negative thoughts and experiences will be identified. In the second phase, the Preparation phase, stabilization techniques such as inducing a “safe place” will be addressed. In the Assessment phase the clinician identifies the components of the target being the tinnitus complaints. The negative belief, emotional and physical sensations about the tinnitus will be made concrete. The patient will rate the amount of distress using the 0–10 (0 = neutral to 10 = the worst disturbance imaginable) Subjective Units of Disturbance Scale (SUDS) and the strength of the desired belief by using the Validity of Cognition scale (VOC) with a range of 1–7 (1 = completely false to 7 = completely true).

In the reprocessing phases, consisting of the Desensitization and Installation phase and Body Scan, the bilateral stimulation will be used to activate the traumatic network. During these phases the clinician will monitor how the patient is processing information associated with the tinnitus. In the last phases, the Closure and Reevaluation phases, the patient is brought to equilibrium, and the distress level and positive treatment effects will be evaluated. Every patient who is randomized in the TRT + EMDR group will receive five 60-min sessions of EMDR over five consecutive weeks.

### Side effects

There are no known side effects of TRT, CBT or EMDR. It is possible that some emotions are triggered and temporarily elevated due to the processing of certain experiences.

### Ethics

Written consent will be obtained from every patient. The Ethical Committee of the Antwerp University Hospital approved the study protocol on 17 October 2016 with protocol number EC UZA 16/35/360.

### Assessment measures

**Fig. 2:**
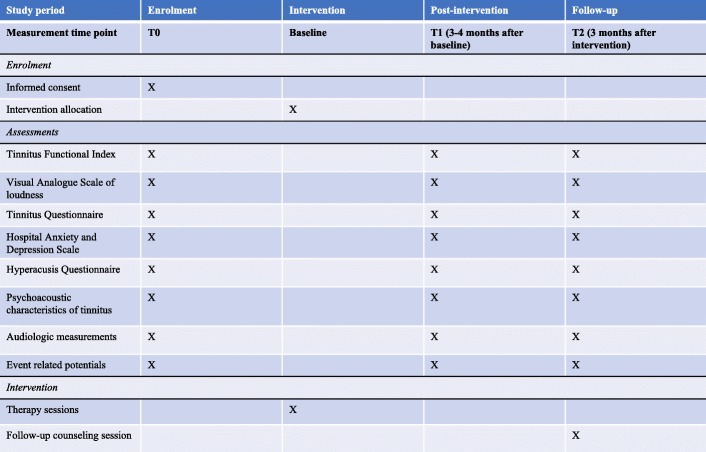
Standard Protocol Items: Recommendations for Interventional Trials (SPIRIT) guideline schedule of enrollment, interventions and assessments for both intervention groups

Subjective and objective assessment measures will be used to assess the effectivity of both interventions. Self-reported assessment measures and questionnaires provide us with important subjective information. To date, tinnitus research is being challenged to provide objective measures as well. By performing ERP assessment we are able to collect objective data. In Fig. 2 we give an oversight of the enrollment, interventions and assessment for the two bimodal therapies.

### Primary outcome measurement

#### Tinnitus Functional Index (TFI)

The TFI is a self-reported questionnaire, consisting of 25 questions, which assesses the impact of tinnitus on patients’ daily lives [35, 36]. The patient answers each question on a Likert scale ranging from 0 to 10. Questions 1 and 3 are expressed in percentages, and the Likert scale ranges from 0 to 100%. The total score is calculated with the mean of all questions. The answers are converted and the total score is expressed as a number between 0 and 100. In addition to the total score, the score of eight subscales can be determined. The subscales are the following: intrusiveness, reduced sense of control, cognitive interference, sleep disturbance, auditory difficulties attributed to tinnitus, interference with relaxation, reduced quality of life and emotional distress.

A decrease in the score on the TFI in the TRT-CBT treatment group versus a decrease in the TFI score in the TRT- EMDR-treatment group is the primary focus of attention in this study.

### Secondary outcome measurements

#### Visual Analogue Scale (VAS) of loudness

The patient scores the mean and maximum loudness of their tinnitus on a scale of 0 (absence of tinnitus) to 100 (as loud as possible, cannot be any louder).

#### Tinnitus questionnaire (TQ)

The TQ is a 52-item, self-rating scale, which differentiates between dimensions of emotional and cognitive distress, intrusiveness, auditory perceptual difficulties, sleep disturbances and somatic complaints. The patient rates the items on a 3-point scale [37].

#### Hospital Anxiety and Depression Scale (HADS)

The HADS consists of 14 questions that assess anxiety and depression [38, 39]. The patient can choose between four answer options for each question. The score for both components is a summation of the scores of all the questions belonging to the subscale. A result greater than 8 suggests the presence of a depression and/or anxiety disorder.

#### Hyperacusis Questionnaire (HQ)

The HQ is a 14-item questionnaire that surveys a patient’s hypersensitivity to sound [40]. There are four answer options for every question: “no,”, “yes a little,” “yes quite a lot” and “yes a lot” A score of 28 is the cut-off for auditory hypersensitivity.

#### Psychoacoustic measurements

Psychoacoustic characteristics, such as frequency of tinnitus, loudness of tinnitus, and residual inhibition will be determined.

The frequency of tinnitus will be determined by means of frequency matching for which a forced-choice technique is applied. The patient must choose between two presented tones or noises until a tone or noise is found that is similar to the patient’s tinnitus. In case of unilateral tinnitus, the contralateral ear is used as the test ear for frequency matching. In case of bilateral tinnitus, the test ear can be chosen arbitrary. The loudness matching will be performed by comparison of the tinnitus to a pure tone or noise in the ipsilateral ear. Finally, the residual inhibition is the suppression of the patient’s tinnitus, measured by presenting a narrowband noise, 15 dBHL louder compared to the tinnitus loudness, in the ipsilateral ear. This procedure can (partially) mask the tinnitus, which is called a (partial) positive result. When the tinnitus becomes louder, it is called rebound. However, some patients do not notice any change.

#### Audiologic measurements

Impedance measurements will be performed in order to exclude active middle-ear pathology, which is an exclusion criterion of the current study.

In order to categorize our patient group, all subjects will undergo an audiometric hearing test according to current clinical standards (International Organization for Standardization (ISO) 8253–1:2010) with a two-channel Interacoustics AC 40 (Interacoustics A/S, Middelfart, Denmark) in a soundproof audiometric booth. A TDH-39 headphone is used as transducer to measure air conduction thresholds of frequencies ranging from 125 Hz to 16 kHz. Bone conduction thresholds will be determined within a range of 250 Hz to 4 kHz.

In addition, Distortion Product Otoacoustic Emissions (DPOAEs) will be measured at baseline using a pair of two pure-tone frequencies (f1 and f2) closely spaced and presented simultaneously at a level of 65 dB Sound Pressure Level (SPL) for f1 and 55 dB SPL for f2 (frequency ratio f2/f1 = 1.22). The largest and most robust distortion product is 2f1-f2 and can be detected in almost all normal ears.

The last conducted test is the speech-in-noise (SPIN) test. The Leuven Intelligibility Sentence Test (LIST) [41] will be performed. An adaptive procedure is used with the noise at a fixed level of 65 dB SPL. Initially, the speech-to-noise ratio is equal to 0 dB Signal to Noise Ratio (SNR), which implies that the noise and the speech are presented equally loud. Subsequently, the intensity of the speech is varied in steps of 2 dB adaptively in a 1-down (when the keywords in the sentence are repeated correctly), 1-up (when the keywords in the sentence are repeated incorrectly) procedure to determine the 50% correct identification point. This point is called the speech reception threshold (SRT), expressed in dB SNR, and will be determined for each ear. A training list for each ear will be conducted before starting the test.

#### ERP

In this study, the emphasis is on the late auditory evoked potentials, which will be induced by use of a classical oddball paradigm. As such, a standard tone of 1 kHz is repeated with a probability of 80% randomly interrupted by an oddball (or infrequent) tone of 2 kHz with a probability of 20%. This paradigm elicits late auditory evoked potentials comprising of P1-N1-P2 and P300 of which the first complex is mostly generated by bottom-up processes in the brain. The later P300 potential requires more top-down cognitive brain processes and can be seen as an expression of higher cognitive sound processing in tinnitus patients. The hypothesis is that, given the continuous auditory processing of the tinnitus signal, the brain may have less capacity left to align to other incoming stimuli, which may be altered by therapy.

The late auditory potentials will be measured prior to the therapy, after the therapy and 3 months later.

### Statistical methods

Data will be analyzed with SPSS statistical software version 20 (SPSS Inc., Chicago, IL, USA). Descriptive analyses as means and standard deviations (SDs) will be used to describe the characteristics of the participants. Results of both intervention arms will be compared using linear mixed models, accounting for the non-independence of observations within the same individual by including random effect terms into the model. The outcome measurements will be entered as dependent variables. In case this outcome variable shows a non-normal distribution, the data will first be logarithmically transformed. Intervention group (TRT + CBT versus TRT + EMDR) and test moment (T0, T1, T2) are entered as fixed effects. The significance level will be set at *p* < 0.05. The effect of bimodal therapy 1 (TRT + CBT) versus bimodal therapy 2 (TRT + EMDR) will be verified.

### Sample size calculation

A sample size calculation was carried out to determine the sample size required to obtain a power of 80% with α = 0.05 in a two-sided, two-sample *t* test. Assuming an average difference of 0.7 SD between both treatment groups – simplifying the calculation by considering the change in outcome between the first and the last point – a power of 80% is reached with minimal sample size of 65 patients per treatment group. To cover possible drop out, 166 patients will be randomized between therapy group TRT + CBT and therapy group TRT + EMDR. The CONSORT guidelines will be followed and the enrollment data will be transferred into the flow diagram.

### Dissemination protocol

According to the Standard Protocol Items: Recommendations for Interventional Trials (SPIRIT) guidelines, the authors declare that data that break the blind will not be presented prior to release of mainline results. Breaking of the blind will occur at the end of the study. A clinical article will be written on the primary and secondary outcomes of the study and will be disseminated regardless of the magnitude or direction of effect. The present trial is not industry initiated; therefore, there is no publication restriction imposed by sponsors. In addition, a full study report with an anonymized participant-level dataset and statistical code for generating the results will be made publicly available no later than 3 years after termination of the study for sharing purposes.

## Discussion

Currently there is no medical or psychotherapeutic treatment that can alleviate chronic, subjective tinnitus. Tinnitus sufferers often hear that they will have to learn to live with this condition. However, for a significant amount of the population the tinnitus causes severe interference with daily functioning and present treatment methods may not provide sufficient relief for these specific patients. For patients experiencing great decrease in their quality of life, EMDR could be an important therapy since it has shown to be effective in the treatment of medically unexplained symptoms such as chronic pain and phantom pain. Seeing the fact that tinnitus is described as a phantom percept, we suggest that EMDR may be of significant value in treating patients with subjective, chronic tinnitus.

The main objective is to evaluate whether the bimodal therapy TRT and EMDR can provide faster and/or more relief from the annoyance caused by the tinnitus sound compared to the bimodal therapy TRT and CBT.

So far there is no clinical trial that uses this combination of methods as a treatment for tinnitus.

Furthermore, we believe this is the first research project that will analyze subjective and objective parameters as outcome measurements in bimodal therapy in the treatment of tinnitus.

### Trial status

To date, patient recruiting is in the final phase. More than 100 patients have been randomized and have ended treatment or are being treated. The end date of study will be June 2019.

## Additional file

Additional file 1: Standard Protocol Items: Recommendations for Interventional Trials (SPIRIT) 2013 Checklist: recommended items to address in a clinical trial protocol and related documents. (DOC 123 kb)

## Abbreviations

AIP: Adaptive Information Processing
CBT: Cognitive behavioral therapy
dBHL: Decibels hearing level
qEEG: Electroencephalography
EMDR: Eye Movement Desensitization Reprocessing
ENT: Ear, Nose and Throat
ERP: Event-related potential
HADS: Hospital Anxiety and Depression Scale
HQ: Hyperacusis Questionnaire
ISO: International Organization for Standardization
MEG: Magnetoencephalography
MML: Minimal masking level
SPIRIT: Standard Protocol Items: Recommendations for Interventional Trials
TFI: Tinnitus Functional Index
TQ: Tinnitus Questionnaire
TRT: Tinnitus Retraining Therapy
VAS: Visual Analogue Scale
